# High Levels of Genetic Differentiation between Ugandan *Glossina fuscipes fuscipes* Populations Separated by Lake Kyoga

**DOI:** 10.1371/journal.pntd.0000242

**Published:** 2008-05-28

**Authors:** Patrick P. Abila, Michel A. Slotman, Aristeidis Parmakelis, Kirstin B. Dion, Alan S. Robinson, Vincent B. Muwanika, John C. K. Enyaru, Loyce M. Lokedi, Serap Aksoy, Adalgisa Caccone

**Affiliations:** 1 National Livestock Resources Research Institute, Tororo, Uganda; 2 Department of Ecology and Evolutionary Biology, Yale University, New Haven, Connecticut, United States of America; 3 Joint FAO/IAEA Programme of Nuclear Techniques in Food and Agriculture, Insect Pest Control Sub-Programme, International Atomic Energy Agency, Vienna, Austria; 4 Institute of Environment and Natural Resources, Makerere University, Kampala, Uganda; 5 Department of Biochemistry, Faculty of Science, Makerere University, Kampala, Uganda; 6 Yale University School of Public Health, New Haven, Connecticut, United States of America; Case Western Reserve University School of Medicine, United States of America

## Abstract

**Background:**

*Glossina fuscipes fuscipes* is the major vector of human African trypanosomiasis, commonly referred to as sleeping sickness, in Uganda. In western and eastern Africa, the disease has distinct clinical manifestations and is caused by two different parasites: *Trypanosoma brucei rhodesiense* and *T. b. gambiense*. Uganda is exceptional in that it harbors both parasites, which are separated by a narrow 160-km belt. This separation is puzzling considering there are no restrictions on the movement of people and animals across this region.

**Methodology and Results:**

We investigated whether genetic heterogeneity of *G. f. fuscipes* vector populations can provide an explanation for this disjunct distribution of the *Trypanosoma* parasites. Therefore, we examined genetic structuring of *G. f. fuscipes* populations across Uganda using newly developed microsatellite markers, as well as mtDNA. Our data show that *G. f. fuscipes* populations are highly structured, with two clearly defined clusters that are separated by Lake Kyoga, located in central Uganda. Interestingly, we did not find a correlation between genetic heterogeneity and the type of *Trypanosoma* parasite transmitted.

**Conclusions:**

The lack of a correlation between genetic structuring of *G. f. fuscipes* populations and the distribution of *T. b. gambiense* and *T. b. rhodesiense* indicates that it is unlikely that genetic heterogeneity of *G. f. fuscipes* populations explains the disjunct distribution of the parasites. These results have important epidemiological implications, suggesting that a fusion of the two disease distributions is unlikely to be prevented by an incompatibility between vector populations and parasite.

## Author Summary

The two types of sleeping sickness in West and East Africa are markedly distinct, require different treatments, and are caused by different parasites. The only country where both parasites are present is Uganda, where they are separated by a narrow 160 km disease-free belt. Because there is no restriction on the movement of humans and animals between the two disease zones, this separation is puzzling. We asked whether this disjunct distribution can be explained by variation within the tsetse fly that is largely responsible for transmitting both diseases in Uganda, *Glossina fuscipes fuscipes*. We therefore examined whether this tsetse subspecies is genetically uniform across Uganda. Our results indicate that *G. f. fusicipes* is not genetically different between the two disease zones, but there are clear genetic differences between northern and southern populations, which are separated by Lake Kyoga. Therefore, it is unlikely that variation in the tsetse fly determines the distribution of the two parasites. This implies that the two diseases may fuse in the near future, which would greatly complicate diagnosis and treatment of sleeping sickness in any potential area of overlap.

## Introduction

Tsetse (Diptera: Glossinidae) are the sole vectors of pathogenic trypanosomes in tropical Africa, where they cause Human African trypanosomiasis (HAT), or sleeping sickness. HAT is a zoonosis caused by the flagellated protozoa *Trypanosoma brucei rhodesiense* in East and Southern Africa and by *T. b. gambiense* in West and Central Africa, with the two diseases separated geographically more or less along the line of the Great Rift Valley [1]. The pathologies of the parasite subspecies are markedly different. Disease resulting from *T. b. rhodesiense* has a rapid onset leading to a fatal condition within the first 6 months of infection, while infection with *T. b. gambiense* produces a chronic condition with long symptom-free periods, which may last several years [2]. It is estimated by the World Health Organization (WHO) that there are still around 100,000 cases of HAT, with 60 million people at risk in 37 countries covering about 40% of Africa [3],[4]. In addition to the human disease-causing parasites, the related species *T. b. brucei*, *T. congolense* and *T. vivax* are responsible for a fatal disease (nagana) in cattle, domestic pigs, and other farm animals. Nagana has restricted agricultural development and nutrient availability and has had a profound economic effect on the continent [5],[6], with an estimated annual economic loss of $4.5 billion US in livestock alone [7].

**Figure 1:**
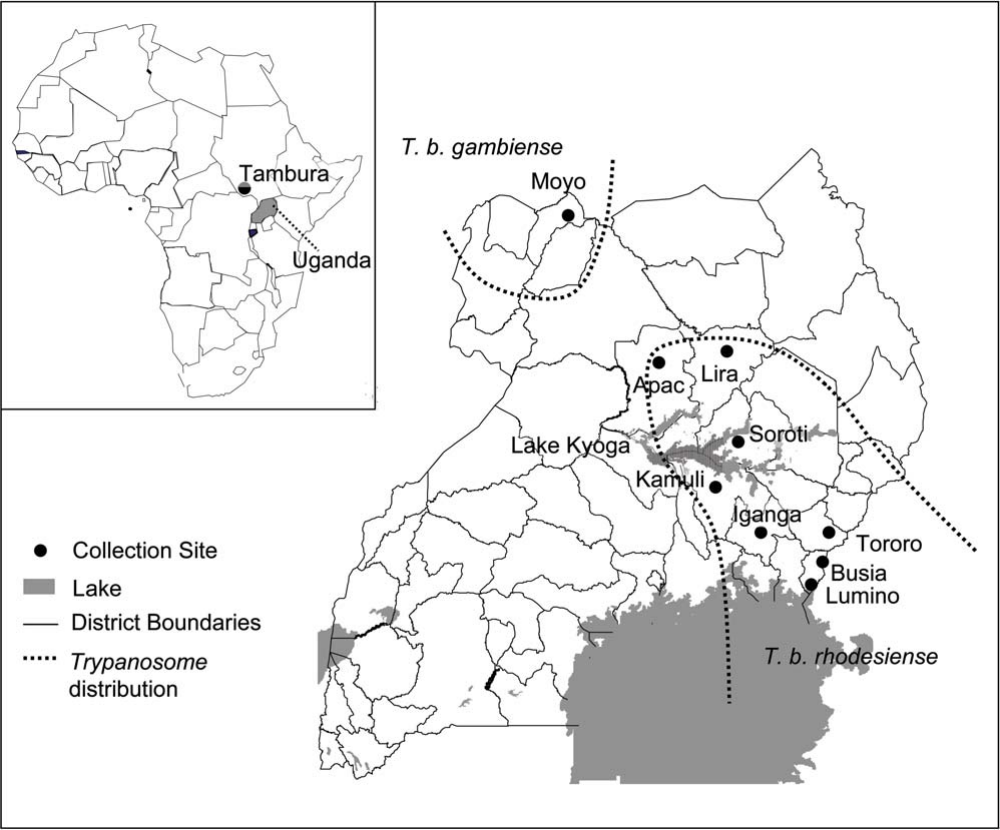
Map of nine sampling locations of *Glossina fuscipes fuscipes* in Uganda, as well as a sampling site in Southern Sudan.

The only country with known foci of infection with both parasites is Uganda, with *T. b. gambiense* present in the north-west and *T. b. rhodesiense* found in the south (Figure 1) [8]. Despite unrestricted movement of cattle and people, *T. b. gambiense* and *T. b. rhodesiense* have maintained a disjunct distribution. However, *T. b. rhodesiense* has recently spread westward into districts previously uninfected, so that only a 160 km belt remains between the two parasites (Figure 1) [9]–[12]. Given the differences in disease pathologies and treatment of the two parasites, combined with the difficulty of timely diagnosis, the coalescence of the distribution of the chronic and acute forms of the disease will pose a critical problem for its control and treatment.

The tsetse flies that are vectors of HAT belong to the genus *Glossina.* This genus is subdivided into three subgenera; *morsitans*, *fusca*, and *palpalis*, consisting of 33 currently recognized species and subspecies [13]. Although all species of tsetse are potential vectors, the major human disease vectors are members of the *palpalis* and *morsitans* complex [14], which constitute riverine + forest and savannah flies, respectively. The *fusca* group is found in forest habitat and contains species that rarely feed on people. While control of savannah species can be sufficiently realized through traditional trapping technologies [15], these are less effective for reducing riverine fly populations.

In Uganda, where tsetse flies are estimated to infest approximately 2/3 of the total land area [16], three major *Glossina* species are present: *G. fuscipes*, *G. pallidipes*, and *G. brevipalpis* [17], belonging to the *palpalis, morsitans* and *fusca* subgenera respectively. As a result of human expansion and habitat reduction, *G. pallidipes* and *G. brevipalpis* populations were greatly reduced by the early 1980's, while *G. fuscipes* population densities have increased steadily [18],[19]. *G. fuscipes* has a wide geographic distribution in sub-Saharan Africa and is comprised of three allopatric subspecies; *G. f. fuscipes, G. f. martinii*, and *G. f. quanzensis*. Of these, *G. f. fuscipes* has the broadest distribution. It is the only subspecies found in Uganda, located at the eastern margin of its range, which extends further east only along the shores of Lake Victoria in Western Kenya. The range of *G. f. fuscipes* extends westward across the central part of the African continent, and includes southern Sudan, Chad, the Central African Republic, the Democratic Republic of Congo (DRC), and Angola. Isolated populations are also present in southwestern Ethiopia and southern Sudan [20].

*G. pallidipes* and *G. brevipalpis*, the two other Ugandan tsetse species, are at low densities and have ranges that include the country's drier forest patches. In contrast, *G. f. fuscipes*, a riverine species, has poor waterproofing abilities and low water reserves. Therefore, the majority of *G. pallidipes* and *G. brevipalpis* habitat is unsuitable for *G. f. fuscipes*. Instead, *G. f. fuscipes* is confined to hydrophytic habitats, such as forested patches along rivers and lacustrine environments [21]. *G. f. fuscipes* habitat extends throughout much of Uganda, including the narrow belt separating the two diseases, whereas this area is unsuitable to *G. pallidipes* and *G. brevipalpis*. Importantly, the latter two species feed mostly on wild animals, whereas *G. f. fuscipes* feeds on the wild and domestic animals that serve as reservoirs for the parasites, as well as humans [22]–[24]. This opportunistic feeding behavior, coupled with a high population density, causes *G. f. fuscipes* to be the most important human disease vector species in Uganda [11],[25].

Population genetic data on a variety of tsetse species, including savannah (*G. morsitans*, *G. pallidipes*, *G. swynnertoni*), forest (*G. palpalis palpalis*), and riverine flies (*G. palpalis gambiensis*) indicate relatively high levels of genetic structuring [13], [26]–[29]. This finding may not be unexpected given the patchy distribution of tsetse populations, even though tsetse have the ability to disperse hundreds of meters daily [30],[31]. Although all studied tsetse show relatively high levels of genetic structuring, indicating low levels of gene flow, in comparison to other tsetse, *G. swynnertoni*, a savannah species from the highland of Tanzania, as well as *G. p. gambiensis*, a riverine species from West Africa, show the highest levels of gene flow. While estimates of gene flow among *G. swynnertoni* populations might be inflated because of a recent genetic expansion [26], those between *G. p. gambiensis* populations are likely to be more accurate and reflect linear dispersal along water bodies bordering its patchy forest habitat [32]–[36].

The high level of genetic structuring observed in various tsetse species suggests that genetic heterogeneity in *G. f. fuscipes* populations could be responsible for the disjunct distributions of *T.b. rhodensiense* and *T.b. gambianse* in Uganda. That is, *G. f. fuscipes* could consist of genetically distinct populations, with the two *Trypanosoma* subspecies adapted to the specific genotypes found in their respective host populations.

Therefore, we used nuclear (microsatellite) and mitochondrial DNA (mtDNA) data to analyze levels and patterns of genetic differentiation between *G. f. fuscipes* populations throughout Uganda, including populations from both the *T. b. rhodesiense* and *T. b. gambiense* diseases belts. These data are not only relevant with respect to the disjunct distribution of the two *Trypanosoma* subspecies, but through a comparison with the structure of other tsetse populations also provide insight into factors responsible for governing tsetse distribution and migration. These findings will contribute to the development and planning of tsetse intervention and disease control strategies.

## Materials and Methods

### Sample Collection

*G. f. fuscipes* specimens were collected from nine locations in Uganda and one location in southern Sudan between March 2004 and August 2005. Five of the Ugandan populations, *i.e.* Kamuli, Tororo, Lumino, Busia, and Iganga, are located south of Lake Kyoga; and four locations; *i.e.* Moyo, Soroti, Lira and Apac are located north of the lake (Figure 1). Moyo and Tambura are from the *T. b. gambiense* disease belt, whereas all other populations are from the *T. b. rhodesiense* disease belt. Samples were collected using non-impregnated biconical traps using standard procedures [37]. Either legs or abdomens were used for DNA extraction. See Table 1 for sample sizes.

**Table 1:** Sample sizes and neutrality test estimates for ten populations of *Glossina fuscipes fuscipes.*

Population	N (Microsatellites)	N (mtDNA)	Fs (mtDNA)*	R_2_ (mtDNA)*	Theta (mtDNA)
Tororo	55.2 (36–67)	35	−2.731	0.064	1.2
Lumino	12.6 (11–13)	12	2.492	0.215	3.6
Iganga	20.4 (13–28)	19	0.103	0.164	2.4
Kamuli	55.8 (32–62)	40	0.468	0.179	1.8
Moyo	36.4 (33–38)	21	−0.144	0.166	2.0
Apac	18.8 (18–20)	15	0.440	0.137	1.4
Soroti	21.2 (19–22)	8	3.850	0.238	9.0
Lira	48 (31–63)	30	1.446	0.173	7.7
Busia	-	11	1.276	0.185	2.5
Tambura (Sudan)	-	11	1.740	0.131	7.3

For microsatellites, N is averaged over 5 loci and values between brackets are minimum and maximum N. Significance level set at 0.01

### Molecular Methods

Extraction of genomic DNA was performed following [38], or using the Easy DNA Kit (Invitrogen). Primers to amplify five microsatellite loci were developed based on clones of a microsatellite enriched library. The library was in *E. coli* (strain DH5 alpha) transformed with recombinant plasmid pUC 19. This library was constructed by the Genetic Identification Services, California, USA, using total genomic DNA extracted from the thoracic muscle of teneral flies from a *G. f. fuscipes* colony maintained at the International Atomic Energy Agency (IAEA) in Seibersdorf, Austria. The colony was established in Seibersdorf in 1986 and originated from flies collected in the Central African Republic. Primers for these loci were as follows: B03For 5′ GGAGGCTATGCTGATGAATG 3′, B03Rev 5′ TGATGCGAAAAAGAGAAACAG 3′, D05For 5′ TTTCCTTCCAGACGAACTG 3′, D05Rev 5′ CTTGGTATGGTCGTACATGG 3′, B05For 5′ CGCGCTTAGCTAGGAAACTC 3′, B05Rev 5′ AACGATTTGCTGTCCTCGAT 3′, D101For 5′ TGCCTTTACACTGCATACTACC 3′ , D101Rev 5′ AAAAAGAGGAGCAATGATGTG 3′, D12For 5′ GTTGATGGTCACACAACATAAG 3′, D12Rev 5′ TCAATGAGGAAAACTGAACTG 3′. Polymerase Chain Reaction (PCR) amplifications were performed using fluorescently labeled forward primers in 20 µl reactions containing 1 µl template DNA, 2 µl 10X PCR buffer, 1 mM of MgCl_2_, 0.5 µM dNTP's, 1 µM of each primer, and 1 unit of AmpliTaq Gold (Applied Biosystems). PCR reactions were performed using the following program: 10 min of denaturation at 94 °C, followed by 35 cycles of 30 sec at 94 °C, 30 sec at 55 °C, and 30 sec at 72 °C. All reactions were followed by a final extension step of 20 min at 72 °C. PCR products were diluted 1/10 and run on an ABI 3730 automated sequencer. Genotype scoring was performed using Genemapper version 3.7 (Applied Biosystems).

PCR amplification of 349 bp of the mtDNA COII gene and 433 bp of the CytB gene using universal invertebrate primer pairs mtD13/mtD15 and mtD26/mtD28 respectively [39] was also achieved. PCRs were performed in 25 µl containing 1 µl of template DNA, 2.5 µl 10X PCR buffer, 0.8 mM dNTP, 2 mM MgCl_2_, 0.4 µM of each primer, 1 µl of BSA and 1 unit of AmpliTaq Gold (Applied Biosystems). Thermal cycler conditions consisted of an initial 10 min denaturation step at 94 °C, followed by 35 cycles of 1 min at 94 °C, 1 min at 48 °C, and 1 min at 72 °C. Reactions were terminated with a final extension time of 5 min at 72 °C. PCR products were purified with the Qiaquick PCR Purification Kit (Qiagen) and sequenced on an ABI 3730 automated sequencer (Applied Biosystems) following standard manufacturer protocols. Sequencing was performed in both the forward and reverse directions to minimize error.

### Microsatellite data analysis

Average heterozygosity and allelic richness for the microsatellite loci were calculated using FSTAT [40]. The program Microchecker [41] was used to determine if null alleles were present in our data set. Tests of Hardy-Weinberg and linkage disequilibrium (10,000 permutations) were performed using Arlequin version 3.1 [42]. Arlequin was also used to perform a locus-by-locus AMOVA of the microsatellite data set in which populations north and south of Lake Kyoga were grouped (10,000 permutations). Additionally, an AMOVA was performed in which the Moyo population, which transmits *T. b. gambiense*, was considered a single group and the other three northern populations, Apac, Lira and Soroti, which transmit *T. b. rhodesiense*, were clustered. *Fst* values between populations were calculated using the *ENA* method implemented in FreeNA [43], which corrects for the presence of null alleles. Because this software only implements bootstrapping over loci to determine significance of *Fst* values, these were also calculated using Arlequin (10,000 permutations). *Fst* values calculated with FreeNA were used to construct a neighbor-joining tree in PAUP version 4.0b10 [44], and to perform a Mantel test to determine if genetic and geographic distances between populations are correlated using Isolation By Distance Web Service version 3.14 (10,000 randomizations) [45]. We used the program Structure [46] to determine the most likely number of clusters (*k*) within our dataset. These analyses were run for 350,000 generations with a burn-in of 100,000. Seven runs were performed for *k* = 1 to 8. This analysis was also performed including only the northern four populations to determine if populations transmitting different *Trypanosoma* parasites are differentiated.

### mtDNA data analysis

Sequence data from COII and CytB were edited with Sequencher 4.2.2. (Gene Codes Corporation) and the data from the two genes were combined for all subsequent analyses. Alignments were performed with Clustal W [47]. MtDNA diversity indices, including the number of haplotypes (H), haplotypic diversity (h), and nucleotide diversity (π), were estimated for each population using DnaSP v. 4.10.9 [48]. An AMOVA, in which populations north and south of Lake Kyoga were grouped, was performed following Excoffier et al. [49] using Arlequin version 3.1 [42]. For this analysis the Tambura (Sudan) population was excluded, but an additional AMOVA was performed in which this population was included as a third group. Additionally, the four northern populations were divided into two clusters, separating Moyo from Apac, Lira and Soroti, and the analysis was repeated. Arlequin was also used to calculate pairwise *Fst* values for the mtDNA data set following Excoffier et al. [42]. These *Fst* values were used to perform a Mantel test for Isolation-by-Distance using Isolation by Distance Web Service version 3.14 (10,000 randomizations) [45]. A haplotype network was constructed using TCS version 1.18.mac software package [50]. The 95% parsimony criterion was used for connecting haplotypes, and all instances of alternative connections were resolved using predictions from coalescent theory as described in Posada and Crandall [51].

To detect departure from selective neutrality and demographic equilibrium, a Fs test [52] and R_2_ test [53] were performed. If neutrality can be assumed and there is no genetic hitchhiking, these are the most powerful available tests to detect historical demographic expansions [53],[54]. DNAsp v. 4.10.9 provides *p-*values based on a coalescent simulation algorithm (10,000 simulations were run). A significant p-value may be caused by violation of any of the assumptions in the null hypotheses; neutrality, constant population size, panmixia, or no recombination. Significant negative departures of these tests are caused by an excess of new mutations resulting from evolutionary forces such as selective sweeps or population expansion. Processes that maintain an excess of old mutations result in positive departures [55]. The mtDNA sequence data have been submitted to Genbank under accession nos EU559605-EU559621 (COII) and EU562262-EU562281 (CytB).

## Results

### Microsatellite data set

Eight Ugandan *G. f. fuscipes* populations from the two disease belts were analyzed using five microsatellite loci. One population, Moyo, transmits *T. b. gambiense*, whereas the other seven populations transmit *T. b. rhodesiense.* Heterozygosity varied greatly between loci and between populations, with Kamuli fixed for a single allele at locus D12, and a heterozygosity of 0.80 for locus D05 in Apac (Table S1). Heterozygosity averaged over all populations was 0.60, 0.54, 0.35, 0.41 and 0.23 for loci D05, B05, D101, B03 and D12, respectively. The number of observed alleles was 11, 5, 6, 13 and 6, respectively. Allelic richness, the number of observed alleles per population corrected for sample size, ranged from 1 to 6.2 (Table S2).

Out of 80 tests for linkage disequilibrium, three were significant after Bonferroni correction. Two of these tests were between locus B05 and D101 (Lira and Iganga), and these two loci also showed significant linkage disequilibrium in Tororo and Soroti before Bonferroni correction. This could indicate that these two loci may be linked and are not fully independent markers. However, in two other populations these two loci were completely unlinked (p = 1). Out of 40 tests of Hardy-Weinberg disequilibrium, only locus D05 in the Tororo population showed a significant excess of homozygotes after Bonferroni corrections. That is, we found no evidence for subdivision within populations.

An analysis using Microchecker detected the possible presence of null alleles in 13 out of 40 tests. Because this can bias estimates of genetic differentiation, *Fst* values were calculated using the *ENA* algorithm [43], which corrects for null alleles, resulting in relatively unbiased *Fst* estimates (Table 2). *Fst* values were also calculated without correcting for null alleles (Table 2) to determine if their presence created a substantial bias. Although, there are some differences between the corrected and uncorrected estimates of genetic differentiation, none were substantial, and no consistent bias was observed.

**Table 2:** Genetic differentiation (*Fst*-values) between eight populations of *G. f. fuscipes* from Uganda based on five microsatellite loci.

	Tororo	Lumino	Iganga	Kamuli	Moyo	Apac	Soroti	Lira
Tororo	-	0.022	0.064	0.104	0.289	0.376	0.221	0.234
Lumino	0.022	-	0	0.062	0.226	0.339	0.178	0.201
Iganga	0.034^*^	0	-	0.031	0.266	0.369	0.218	0.226
Kamuli	0.103^***^	0.033	0.010	-	0.395	0.517	0.382	0.340
Moyo	0.337^***^	0.278^***^	0.219^***^	0.419^***^	-	0.112	0.103	0.064
Apac	0.443^***^	0.397^***^	0.377^***^	0.534^***^	0.128^***^	-	0.089	0.110
Soroti	0.266^***^	0.225^***^	0.187^***^	0.388^***^	0.112^***^	0.104^***^	-	0.084
Lira	0.266^***^	0.222^***^	0.239^***^	0.348^***^	0.041	0.084^***^	0.055^***^	-

Above diagonal: corrected for null alleles, below diagonal: not corrected for null alleles. ^*^ p<0.05, ^**^p<0.01, ^***^ P<0.001.

**Figure 2:**
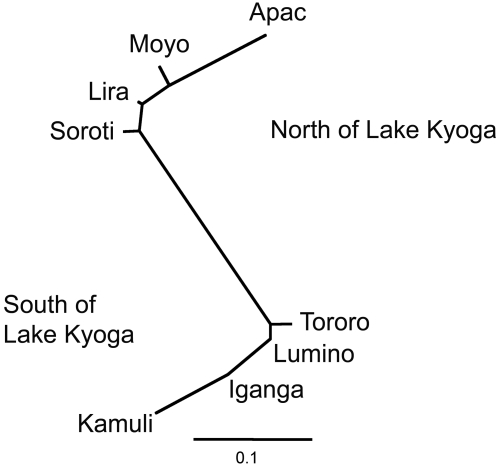
Neighbor-Joining tree based on microsatellite pairwise *Fst* values between Ugandan populations of *Glossina fuscipes fuscipes*.

The FreeNA software implements a significance test based on bootstrapping over loci, resulting in a very weak test. Our use of relatively few loci further reduced the test's power. No significant differentiation was observed between populations based on these tests. However, the much more powerful permutation tests implemented in Arlequin using the uncorrected data set detected highly significant differentiation in most pair-wise comparisons between populations. Importantly, genetic differentiation between populations from opposite sides of the Lake was always larger than between populations from the same side, and the few non-significant pairwise *Fst* values are between populations from the same side of Lake Kyoga. This pattern is also clear from the neighbor-joining tree constructed using these *Fst* values (Figure 2), which visualizes the genetic differentiation between populations. The populations from opposite sides of Lake Kyoga, henceforth referred to as northern vs. southern populations, cluster relatively close together, with a larger genetic differentiation between the two groups.

**Figure 3:**
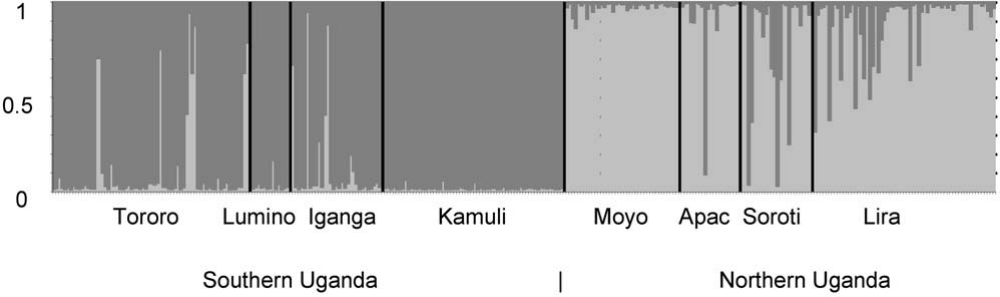
Individual bayesian assignment probabilities for *k* = 2 for 9 populations of *Glossina fuscipes fuscipes* from Uganda. Each vertical bar represents a single individual.

A population clustering analysis using the program Structure clearly indicated that populations north and south of Lake Kyoga indeed belong to two separate clusters. For *k* = 1 the LR score = −2860.6, whereas the LR score = −2324.9 for *k* = 2, stabilizing between −2316.9 to −2371.5 for *k* = 3 to 8. Therefore, the Structure analyses did not detect any additional substructure within the northern or southern groupings. In Figure 3, the probability of each individual belonging to one of the two clusters is presented. In all, 93.5% of northern individuals and 93.7% of southern individuals were assigned to their respective group. This clustering of *G. fuscipes* populations is also clear from an AMOVA based on the microsatellite data (Table 3). Between group differences account for 26.9% of the variation, whereas differences within groups explain only 5.9%.

**Table 3:** Results of AMOVA grouping populations north and south of Lake Kyoga.

	% of variation		
	Microsatellites	mtDNA^a^	mtDNA^b^
Among Groups	26.93	71.74	70.11
Among Populations within Groups	5.94	8.30	8.30
Within Populations	67.1	19.96	21.59

a Excluding Tambura (Sudan), ^b^ Including Tambura (Sudan) as a third group.

We also performed a separate clustering analysis including only the northern populations. This was done to examine whether any further sub-structuring was present within this group, in which populations differ in the *Trypanosoma* parasite species they transmit. This analysis did not detect any additional sub-structuring within the northern group (results not presented).

A Mantel test of isolation-by-distance based on Slatkin's linearized *Fst* values [56] showed no significant correlation between genetic differentiation and geographic distance (p = 0.10) (see Figure S1).

### MtDNA data set

A total of 782 bp from the CytB and COII genes were obtained for 202 *G. f. fuscipes* individuals belonging to nine populations. Two of these, Moyo and Tambura transmit *T. b. gambiense*, whereas the other seven populations transmit *T. b. rhodesiense.* We observed a total of 37 different haplotypes and haplotypic diversity within populations ranged from 0.552 to 0.830, with an overall haplotypic diversity of 0.931 (Table S2). Nucleotide diversity (π) within populations ranged from 0.0016 to 0.0116, with an overall nucleotide diversity of 0.0130 (Table S2).

**Figure 4:**
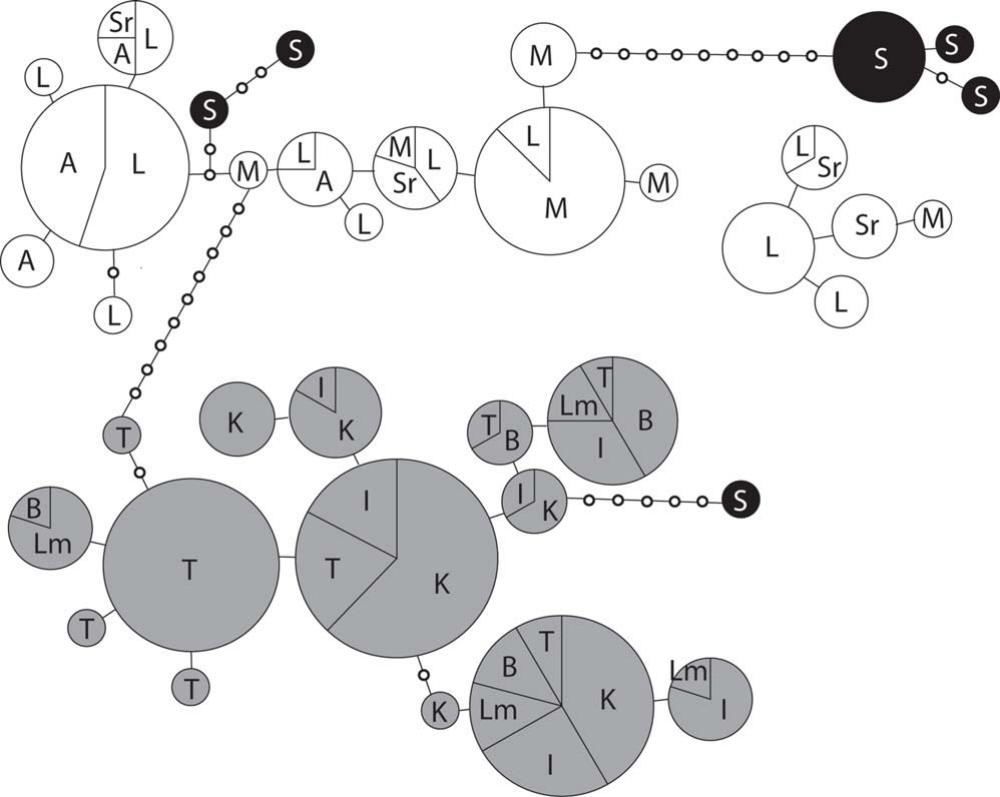
TCS minimum spanning haplotype network based on 782 bp of concatenated COII and Cyt b mtDNA fragments. Circles are proportional to the total number of individuals sharing each haplotype, while slices are proportional to the number of individuals per population carrying a particular haplotype. Ugandan populations south of Lake Kyoga (T = Tororo, Lm = Lumino, I = Iganga, K = Kamuli, B = Busia) are shown in gray, Ugandan populations north of Lake Kyoga (M = Moyo, L = Lira, A = Apac, Sr = Soroti) are shown in white, and the Sudan population (S = Tambura) is shown in black.

The TCS haplotype network shows a clear distinction between populations from the north and south of Lake Kyoga (Figure 4). No haplotypes are shared between these two groups and haplotypes from both groups of populations are separated by a minimum of 10 substitutions. One group of northern haplotypes could not be connected to the main network using the 95% parsimony criterion, however, if this criterion was relaxed to 90% these haplotypes connected to the northern group with a minimum of 13 substitutions. Alternative connections were removed following Posada and Crandall [51]. In one instance, the choice between two alternative connections within the northern group was dubious, but this did not affect the topology of the network with respect to the grouping of northern and southern populations. Although most Sudan haplotypes cluster with northern Uganda samples, as expected based on geography, one Sudanese haplotype surprisingly falls within the southern group.

*Fst* values based on the mtDNA data set between almost all populations were highly significant (Table 4). Within the southern group *Fst* values ranged between 0.010 and 0.504. Within the northern group, *Fst* values ranged between 0.131 and 0.600, and between the northern and southern group *Fst* values ranged from 0.642 to 0.911.

**Table 4:** Genetic differentiation (*Fst*-values) between nine populations of *G. f. fuscipes* from Uganda and one population from Sudan (Tambura) based on COII and CytB mtDNA sequences.

	Tororo	Lumino	Iganga	Kamuli	Busia	Moyo	Apac	Soroti	Lira	Tambura
Tororo	-									
Lumino	0.304^***^	-								
Iganga	0.437^***^	0.075	-							
Kamuli	0.302^***^	0.197^**^	0.134^**^	-						
Busia	0.504^***^	0.010	0.165^*^	0.329^***^	-					
Moyo	0.886^***^	0.834^***^	0.859^***^	0.870^***^	0.862^***^	-				
Apac	0.911^***^	0.858^***^	0.882^***^	0.891^***^	0.890^***^	0.504^***^	-			
Soroti	0.829^***^	0.698^***^	0.771^***^	0.826^***^	0.729^***^	0.581^***^	0.600^***^	-		
Lira	0.722^***^	0.642^***^	0.688^***^	0.733^***^	0.663^***^	0.274^***^	0.168^*^	0.131^*^	-	
Tambura	0.836^***^	0.721^***^	0.773^***^	0.825^***^	0.750^***^	0.633^***^	0.645^***^	0.542^***^	0.464^***^	-

An AMOVA grouping northern and southern populations also clearly indicated this large differentiation between the northern and southern groups (Table 3). Differences between the two groups accounted for 71.74 % of the observed variation, whereas difference between populations within groups accounted only 8.30% of the variation. Including the Sudan population (Tambura) as a third group did not change these results markedly (Table 3).

In contrast to the microsatellite data set, a significant correlation between the geographic distance and linearized *Fst* values between populations was found for the mtDNA data set (p = 0.020) (see Figure S1). No departure from selective neutrality and demographic equilibrium (Table 1) was detected for any population using the Fs and R_2_ tests.

## Discussion

Both the microsatellite and mtDNA data analyses revealed high levels of differentiation between the studied *G. f. fuscipes* populations. Almost all pair-wise comparisons of *Fst* values were significant (Table 2 and 4), indicating some restriction in gene flow between populations. However, the most striking result, which is indicated by both the mtDNA and microsatellite data, is the strong differentiation between the populations north and south of Lake Kyoga. Interestingly, this north-south structuring of *G. f. fuscipes* populations does not coincide with the distribution of *T. b. rhodesiense* and *T. b. gambiense* (Figure 1). Our analyses indicate that the Moyo (microsatellite and mtDNA) and Tambura (mtDNA only) populations lying in the *T. b. gambiense* belt are no more differentiated from the northern populations (Apac, Lira, and Soroti) lying in the *T. b. rhodesiense* belt, as those are from each other. That is, although populations within the northern and southern clusters are in most cases significantly differentiated, we found no evidence of genetic differentiation between tsetse transmitting *T. b. gambiense* vs. *T. b. rhodesiense,* other than would be expected based on their geographic separation.

Our analyses indicated that the microsatellite data set included null alleles. This could have affected the data analysis and have lead to an overestimation of genetic differentiation. However, a comparison of *Fst* values using a method that does not take into account the presence of null alleles vs. the *ENA* method, indicates that no substantial bias was introduced. Additionally, the number of microsatellite loci included in the study was rather small, and heterozygosity was low at locus D12. Therefore, the power of the microsatellite analysis was low, and finer scale patterns of population structuring probably could not be detected in this study. However, the fact that even with this low power we observe highly significant clustering of populations north and south of Lake Kyoga, combined with the observation that the mtDNA data set shows exactly the same pattern, clearly indicates that *G. f. fuscipes* in Uganda is subdivided into at least two distinct clusters with very limited gene flow between them. The forces that maintain the separation of these lineages seem to have been in place for some time since 10 fixed substitutions are present between northern and southern Ugandan populations.

The levels of genetic variation observed for both the mtDNA and the microsatellite markers between *G. f. fuscipes* populations are comparable to those for other *Glossina* species and subspecies. Populations of savannah species, such as *G. morsitans* and *G. pallidipes*, tend to be substantially structured. This is consistent with the patchy distribution of most tsetse populations [27], [29], [35], [57]–[60], but at odds with results from ecological work that suggest a rate of population expansion of about 7 km/year [31],[61],[62]. This high degree of genetic structuring, despite a high dispersal capacity, is thought to be due to dramatic reductions in tsetse population sizes in recent times. This was caused by the rinderpest epidemic in the late 1890s, which killed over 90% of livestock, followed by additional epidemic episodes in the early part of the 20^th^ century, as well as more recent HAT control measures [58],[59],[63],[64]. Since the early 20^th^ century, after episodes of the rinderpest epidemics ceased, tsetse populations have rebounded, and are expanding from highly scattered relict populations. Population recovery has also been assisted by reduced control efforts due to unstable political and economic conditions.

In contrast to the strong genetic structuring found in savannah and forest tsetse species, the riverine species *G. p. gambiensis* in West Africa has low levels of genetic differentiation between populations [32],[33],[35],[36]. This species lives in humid savannah and can easily disperse through the forests along riverbanks. Such linear dispersal through suitable habitat was also observed for *G. palpalis*, for which mark-release-recapture studies indicate that it can disperse up to 21 km in 5 days along gallery forests [32],[65], but only 8 km along rivers with bare banks [66]. However, gene flow among *G. gambiensis* populations seems to occur not only within single river systems, but also among populations distributed in the different river basins of Mali [67]. This species seems to be expanding or contracting its populations in a pattern that follows the seasonal fluctuations of water level and temperature, resulting in seasonal fusions of the populations.

While *G. p. gambiensis* flies experience high levels of gene flow, suggesting that an isolation-by-distance (IBD) model may best explain their population structure, for a few savannah species the correlation between genetic differentiation and geographic distance was weak, suggesting that an island model, rather than IBD, may best describe the population structure of these tsetse species [29],[59]. In our study we found a significant correlation between genetic and geographic distance when we analyzed all Uganda populations using the mtDNA data set (Figure S1). However, this does not necessarily imply that an IBD model best describes the causal factors associated with the spatial distributions of these populations. The observed IBD pattern is most likely an artifact of the genetic structure caused by Lake Kyoga. That is, the average geographic distance between populations on the two sides of the lake is larger than the average geographic distance between populations on the same side. If more populations were available, a more appropriate test would be to include only populations from either the northern or southern cluster. This issue will be explored in more detail when denser geographic sampling becomes available.

While Lake Kyoga is the main factor in the genetic structuring of Ugandan *G. f. fuscipes* populations, the limited gene flow between populations within the northern and southern group suggests that the patchy distribution of *G. f. fuscipes* populations likewise plays a role in shaping the population structure of these vectors. In this regard, the riverine *G. f. fuscipes* is more similar to the savannah species *G. morsitans* and *G. pallidipes*, than it is to the other riverine species *G. p. gambiensis*. This observation is also supported by Krafsur et al [68], who concluded that the dispersal tendencies of *G. f. fuscipes* are either overestimated, or thwarted by unapparent environmental circumstances in the habitats interspersing the populations included in their study. Consequently, forces of genetic drift in East African *G. f. fuscipes* are much stronger than gene flow.

It is worth noting that the presence of 10 fixed differences in the mtDNA, with the exception of a single Sudanese haplotype, implies an (almost) complete absence of gene flow between the northern and southern populations. However, for the microsatellite makers, even though substantial differences in allele frequencies were observed between northern and southern populations, and each locus carried at least some alleles that were unique to either the north or south, no fixed differences were found. This discrepancy between the mtDNA and microsatellites could indicate a difference in dispersal between males and females. If dispersal is limited to males, fixed differences could accumulate in the maternally inherited mtDNA, whereas even a low number of migrating males would prevent the accumulation of fixed differences in the nuclear microsatellites. However, the lack of fixed differences in the microsatellite markers may also reflect a bias in the loci studied, as variability was one of the criteria for selecting the loci used for this study. Alternatively, the fast, step-wise mode of evolution of microsatellites with its tendency to create homoplasies could explain the lack of fixed microsatellite differences between the north and south.

Within the nine Ugandan populations, both microsatellite and mtDNA data suggest that genetic diversity is relatively high with no evidence of genetic sub-structuring (Table S1, S2). Given that levels of genetic diversity are directly related to effective population size, this suggests that *G. f. fuscipes* population sizes in Uganda may be substantial.

Although there is evidence of sub-structuring within single populations for some forest populations of *G. p. palpalis* in western Africa [34], results from other tsetse species suggest that single locations tend to have genetically homogeneous populations [27],[57]. A recent report on mtDNA variation in three *G. f. fuscipes* populations from the border region between Uganda and Kenya also indicates that single locations have genetically homogeneous *G. f. fuscipes* populations [68]. The population genetic parameters (H, h, theta) we report for *G. f. fuscipes* populations based on the mtDNA diversity (Table 1, Table S2) are comparable to those reported by Krafsur et al. [68]. Our estimates of both mtDNA and microsatellite variation (Table S1) are similar, although at the high end, to those reported for savannah or riverine tsetse species (see Table 5). However, genetic diversity estimates for southern Africa populations of both savannah and forest tsetse species tend to be substantially lower [26],[27],[29],[58]. This is thought to be the result of a dramatic reduction in tsetse population sizes due to the rinderpest epidemic of the late 1890s. This epidemic affected the southern regions of the African continent more severely than others [63],[64]. The level of genetic variation observed in our study indicates that *G. f. fuscipes* in Uganda, like other tsetse from central and western Africa, does not appear to have been severely affected by this event. Furthermore, we found no evidence for bottlenecks or recent population expansions in Ugandan *G. f. fuscipes* populations. This is in congruence with data collected by Krafsur et al [68].

**Table 5:** Population genetic parameters estimated for various *Glossina* taxa.

	mtDNA				
Taxon	No of Populations	H	H	Fst	Reference
*G. m. morsitans* (s)	6	23	0.63	0.09	[60]
*G. m. centralis* (s)	7	7	0.54	0.87	[58]
*G. m. submorsitans* (s)	7	26	0.89	0.35	[71]
*G. pallidipes* (s)	20	26	0.63	0.48	[59]
*G. palpalis gambiensis* (r)	13	9	0.18	0.68	[67]
*G. pallidipes* (s)	21	39	0.42	0.51	[26]
*G. m. morsitans* (s)	7	33	0.81	0.15	[29]
*G. f. fuscipes* (r)	3	21	0.84	0.28	[68]
*G. swynnertoni* (f)	8	17	0.59	0.04	[26]
*G. f. fuscipes* (r)	10	37	0.74	0.59	This study
	Microsatellites				
	No of Populations	No of loci	No of alleles	Fst	Reference
*G. m. morsitans* (s)	6	5	53	0.19	[72]
*G. m. centralis* (s)	7	6	53	0.18	[58]
*G. m. submorsitans* (s)	7	6	49	0.17	[72]
*G. pallidipes* (s)	11	3	18	0.31	[73]
*G. pallidipes* (s)	21	8	214	0.18	[27]
*G. m. morsitans* (s)	9	7	200	0.13	[29]
*G. f. fuscipes* (r)	8	5	41	0.22	This study

s: savannah species, r: riverine species, f: forest species.

Various tsetse populations have been shown to carry infections of the endosymbiont *Wolbachia* [69],[70]. This symbiont, which has infected a wide-range of invertebrate hosts, can cause a variety of reproductive abnormalities, one of which is termed cytoplasmic incompatibility (CI) and results in death early in embryogenesis. In an incompatible cross, the sperm enters the egg but does not successfully contribute its genetic material to the potential zygote, and in most species none or very few eggs hatch. Different strains of *Wolbachia* have been shown to generate such incompatibility. Preliminary studies of *G. f. fuscipes* in Uganda indicate the presence of *Wolbachia* infections (Aksoy, unpublished data). These infections also have the propensity to influence population structure. Future studies on the identification of the *Wolbachia* strains present in the Northern and Southern *G. f. fuscipes* populations can provide additional information on the genetic differentiation between them.

Knowledge on the population structure of tsetse can provide specific guidance on the design of the most effective and economic vector control efforts, as well as on the sustainability of the control efforts. For example, the trapping systems are most effective if the genetic data shows the presence of highly structured populations in the target areas, indicating a minimal risk of re-invasion. Information on genetic differentiation also provides guidance to ongoing control projects as to where the most vulnerable populations reside, and where special effort needs to be given to incorporate physical barriers to prevent reinvasions. For example, our data indicate that tsetse control on either side of Lake Kyoga, is not likely to be affected by migration across or around the lake.

These results also have at least two important epidemiological implications. First, from the vector point of view there is no genome-wide genetic discontinuity at putatively neutral loci across *G. f. fuscipes* populations that can explain the existence of an historical break in the *Trypanosoma* distributions. This separation remains puzzling given unrestricted movement of animals and people across this region. Second, and of more immediate concern, given the narrow and progressively reducing corridor that separates the two diseases, our results imply that a fusion of the *T. b. rhodesiense* and *T. b. gambiense* ranges, currently less than 120 km apart, is unlikely to be prevented by genetic incompatibilities between vector and parasites. Our data suggest that the genetic structuring found among *G. f. fuscipes* Ugandan populations is more likely to reflect past geological and/or biogeographic events, and is not correlated with the subspecies of *Trypanosoma* parasite they transmit.

## Supporting Information

Table S1 Heterozygosity and allelic richness for five microsatellite loci in eight *G. f. fuscipes* populations from Uganda. *H* = Heterozygosity. A.R. = Allelic Richness.
(0.07 MB DOC)

Table S2 Measures of mtDNA diversity in *G. f. fuscipes* populations. n = mtDNA sample size, H = number of haplotypes, h = haplotypic diversity, π = nucleotide diversity (multiplied by 100).
(0.06 MB DOC)

Figure S1 A: Geographic distance vs pairwise linearized Fst values for populations of *G. f. fuscipes* based on the microsatellite data set. B: Geographic distance vs pairwise linearized Fst values for populations of *G. f. fuscipes* based on the mtDNA data set.
(2.41 MB TIF)
