# Correction: High Levels of Genetic Differentiation between Ugandan *Glossina fuscipes fuscipes* Populations Separated by Lake Kyoga

**DOI:** 10.1371/annotation/bc182916-f740-4366-b50e-80410649f197

**Published:** 2008-06-23

**Authors:** Patrick P. Abila, Michel A. Slotman, Aristeidis Parmakelis, Kirstin B. Dion, Alan S. Robinson, Vincent B. Muwanika, John C. K. Enyaru, Loyce M. A. Okedi, Serap Aksoy, Adalgisa Caccone

The eighth author's name was misspelled. The correct name is: Loyce M. A. Okedi. 

