# Supplementary material for: High Levels of Genetic Differentiation between Ugandan Glossina fuscipes fuscipes Populations Separated by Lake Kyoga
Source: PLoS Negl Trop Dis. 2008 May 28;2(5):e242. doi: 10.1371/journal.pntd.0000242 (PMC2386243; doi:10.1371/journal.pntd.0000242)
Supplement: Table S1 — Heterozygosity and allelic richness for five microsatellite loci in eight G. f. fuscipes populations from Uganda. H = Heterozygosity. A.R. = Allelic Richness. (0.07 MB DOC) [file pntd.0000242.s001.doc]

**Table S1** Heterozygosity and allelic richness for five microsatellite loci in eight *G. f. fuscipes* populations from Uganda.

|  | D05 | | B05 | | D101 | | B03 | | D12 | |
| --- | --- | --- | --- | --- | --- | --- | --- | --- | --- | --- |
|  | *H* | *A.R.* | *H* | *A.R.* | *H* | *A.R.* | *H* | *A.R.* | *H* | *A.R.* |
| Tororo | 0.47 | 2.88 | 0.46 | 2.16 | 0.51 | 2.00 | 0.19 | 2.25 | 0.20 | 1.96 |
| Iganga | 0.54 | 2.79 | 0.52 | 2.65 | 0.53 | 3.00 | 0.11 | 1.83 | 0.07 | 1.73 |
| Lumino | 0.50 | 2.00 | 0.51 | 2.00 | 0.50 | 2.00 | 0.33 | 2.00 | 0.26 | 2.00 |
| Kamuli | 0.40 | 2.62 | 0.48 | 2.00 | 0.37 | 2.18 | 0.02 | 1.18 | 0 | 1.00 |
| Moyo | 0.58 | 4.02 | 0.64 | 3.48 | 0.50 | 3.29 | 0.78 | 6.20 | 0.34 | 2.79 |
| Apac | 0.80 | 5.80 | 0.53 | 2.86 | 0.05 | 1.55 | 0.57 | 5.06 | 0.29 | 2.87 |
| Soroti | 0.75 | 4.70 | 0.59 | 2.99 | 0.17 | 1.95 | 0.72 | 4.47 | 0.25 | 2.55 |
| Lira | 0.76 | 5.20 | 0.61 | 2.98 | 0.20 | 1.96 | 0.53 | 4.15 | 0.39 | 3.07 |

*H =* Heterozygosity. A.R. = Allelic Richness.
