# Supplementary material for: High Levels of Genetic Differentiation between Ugandan Glossina fuscipes fuscipes Populations Separated by Lake Kyoga
Source: PLoS Negl Trop Dis. 2008 May 28;2(5):e242. doi: 10.1371/journal.pntd.0000242 (PMC2386243; doi:10.1371/journal.pntd.0000242)
Supplement: Table S2 — Measures of mtDNA diversity in G. f. fuscipes populations. n = mtDNA sample size, H = number of haplotypes, h = haplotypic diversity, π = nucleotide diversity (multiplied by 100). (0.06 MB DOC) [file pntd.0000242.s002.doc]

**Table S2** Measures of mtDNA diversity in *G. f. fuscipes* populations.

|  | *n* | *H* | *h* | *π* |
| --- | --- | --- | --- | --- |
| Tororo | 35 | 8 | 0.585 | 0.160 |
| Iganga | 19 | 6 | 0.813 | 0.303 |
| Lumino | 12 | 4 | 0.773 | 0.457 |
| Kamuli | 40 | 6 | 0.724 | 0.230 |
| Busia | 11 | 4 | 0.745 | 0.317 |
| Moyo | 21 | 6 | 0.552 | 0.259 |
| Apac | 15 | 4 | 0.619 | 0.180 |
| Soroti | 8 | 4 | 0.821 | 1.161 |
| Lira | 30 | 11 | 0.830 | 0.999 |
| Tambura (Sudan) | 11 | 6 | 0.727 | 0.950 |
| Total | 202 | 37 | 0.931 | 1.302 |

n = mtDNA sample size, H = number of haplotypes, h= haplotypic diversity,

π = nucleotide diversity (multiplied by 100).
