# Supplementary figures and images for: High Levels of Genetic Differentiation between Ugandan Glossina fuscipes fuscipes Populations Separated by Lake Kyoga
Source: PLoS Negl Trop Dis. 2008 May 28;2(5):e242. doi: 10.1371/journal.pntd.0000242 (PMC2386243; doi:10.1371/journal.pntd.0000242)

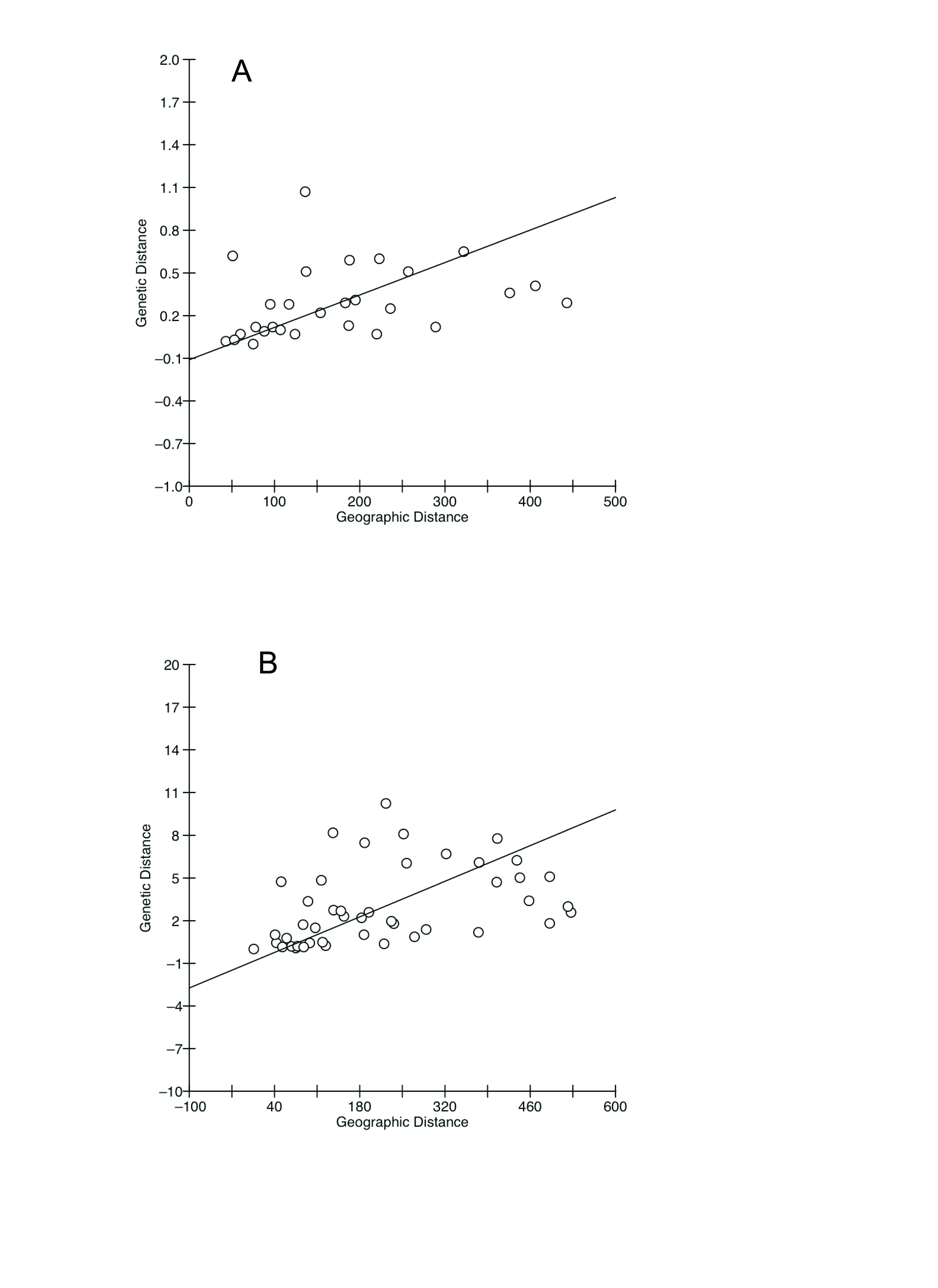

Supplement: Figure S1 — A: Geographic distance vs pairwise linearized Fst values for populations of G. f. fuscipes based on the microsatellite data set. B: Geographic distance vs pairwise linearized Fst values for populations of G. f. fuscipes based on the mtDNA data set. (2.41 MB TIF) [file pntd.0000242.s003.tif]
